# Molecular and Functional Assessment of *TSC1* and *TSC2* in Individuals with Tuberous Sclerosis Complex

**DOI:** 10.3390/genes15111432

**Published:** 2024-11-03

**Authors:** Luiz Gustavo Dufner-Almeida, Laís F. M. Cardozo, Mariana R. Schwind, Danielly Carvalho, Juliana Paula G. Almeida, Andrea Maria Cappellano, Thiago G. P. Alegria, Santoesha Nanhoe, Mark Nellist, Maria Rita Passos-Bueno, Silvana Chiavegatto, Nasjla S. Silva, Sérgio Rosemberg, Ana Paula A. Pereira, Sérgio Antônio Antoniuk, Luciana A. Haddad

**Affiliations:** 1 Human Genome and Stem Cell Research Center, Department of Genetics and Evolutionary Biology, Instituto de Biociências, Universidade de São Paulo, São Paulo 05508-090, Brazil; 2 Department of Clinical Genetics, Erasmus Medical Center, 3015 Rotterdam, The Netherlands; 3 Pediatric Neurology Center, Department of Pediatrics, Hospital de Clínicas, Universidade Federal do Paraná, Curitiba 80060-900, Brazilantoniuk@uol.com.br (S.A.A.); 4 Division of Neurology, Department of Pediatrics, Santa Casa de Misericórdia, São Paulo 01221-010, Brazil; 5 Grupo de Apoio ao Adolescente e à Criança com Câncer, Instituto de Oncologia Pediátrica, Universidade Federal de São Paulo, São Paulo 04039-001, Brazil; 6 Department of Pharmacology, Instituto de Ciências Biomédicas, Universidade de São Paulo, São Paulo 05508-000, Brazil; 7 Department of Psychiatry, Instituto de Psiquiatria, Faculdade de Medicina da Universidade de São Paulo, São Paulo 05403-903, Brazil; 8 Department of Psychology, Universidade Federal do Paraná, Curitiba 80060-000, Brazil

**Keywords:** tuberous sclerosis complex, neurodevelopmental disorder, *TSC1*, *TSC2*, missense variant, splicing variant, Alu repeat, pathogenic alteration

## Abstract

Tuberous sclerosis complex (TSC) is an autosomal dominant neurodevelopmental disorder and multisystem disease caused by pathogenic DNA alterations in the *TSC1* and *TSC2* tumor suppressor genes. A molecular genetic diagnosis of TSC confirms the clinical diagnosis, facilitating the implementation of appropriate care and surveillance. *TSC1* and *TSC2* encode the core components of the TSC1/2 complex (TSC1/2), a negative regulator of the mechanistic target of rapamycin (MTOR) complex 1 (TORC1). Functional analysis of the effects of *TSC1* and *TSC2* variants on TORC1 activity can help establish variant pathogenicity. We searched for pathogenic alterations to *TSC1* and *TSC2* in DNA isolated from 116 individuals with a definite clinical diagnosis of TSC. Missense variants and in-frame deletions were functionally assessed. Pathogenic DNA alterations were identified in 106 cases (91%); 18 (17%) in *TSC1* and 88 (83%) in *TSC2*. Of these, 35 were novel. Disruption of TSC1/2 activity was demonstrated for seven *TSC2* variants. Molecular diagnostics confirms the clinical diagnosis of TSC in a large proportion of cases. Functional assessment can help establish variant pathogenicity and is a useful adjunct to DNA analysis.

## 1. Introduction

Tuberous sclerosis complex (TSC) is a neurodevelopmental disorder and multisystem disease with autosomal dominant inheritance, characterized by brain hamartia (cortical tubers and heterotopic neurons) and hamartomas in multiple organs [1,2]. TSC affects 1:6000–10,000 individuals [1,3,4,5] and is caused by inactivating mutations to the *TSC1* or *TSC2* tumor suppressor genes [6,7]. *TSC1* (NG_012386.1, MIM#605284) maps to 9q34.1, comprises 23 exons, and encodes the TSC1 protein, hamartin (NP_000359.1). *TSC2* (NG_005895.1, MIM#191092) maps to 16p13.3, consists of 42 exons, and encodes the TSC2 protein, tuberin (NP_000539.2). TSC1 and TSC2 are the core components of the TSC molecular complex (TSC1/2), a critical negative regulator of the mechanistic target of rapamycin (mTOR) complex 1 (TORC1). The TSC1/2 is a GTPase-activating protein (GAP) specific for the small GTPase Ras homologue enriched in brain (RHEB). Inactivation of the TSC1/2 results in increased levels of RHEB-GTP, activation of TORC1 kinase activity, and the phosphorylation of downstream TORC1 targets, including p70 S6 kinase (S6K), thus leading to up-regulation of anabolic metabolism and excessive cell growth [8,9,10].

TSC diagnostics has evolved since Gómez first established a broad set of clinical diagnostic criteria [1,11]. In 2012, the International TSC Clinical Consensus Group updated the clinical criteria for TSC and proposed the genetic diagnostic criterion, whereby detection of a pathogenic DNA variant in either the *TSC1* or *TSC2* gene in normal tissue is sufficient for a definite diagnosis of TSC if it prevents protein synthesis or hyperactivates TORC1 according to a functional assay [1,2]. Genetic testing can confirm the clinical definite and possible diagnoses of TSC, allowing early implementation of clinical surveillance and appropriate treatment [2].

Current molecular diagnostic tests identify a pathogenic *TSC1* or *TSC2* variant in nearly 90% of patients with a definite clinical diagnosis of TSC [12], and studies indicate that clinically diagnosed TSC patients with ‘no mutation identified’ (NMI) are most likely to carry either a somatic, mosaic mutation, or a deep intronic variant that affects splicing [13,14,15,16,17,18,19]. In some cases, variants of uncertain clinical significance (VUSs) are identified in individuals with TSC. Functional assessment of the effects of DNA alterations on pre-mRNA splicing and/or TSC1/2 activity can help establish pathogenicity and provide information on how DNA alterations affect the formation, stability, and activity of the TSC1/2.

We conducted a descriptive study on molecular alterations to *TSC1* and *TSC2* in a cohort of 116 Brazilian individuals who had previously received a definite clinical diagnosis of TSC. In 106 cases (91%), a pathogenic *TSC1* or *TSC2* alteration was identified, including single nucleotide changes and large genomic rearrangements. To support pathogenicity, we evaluated the effects of specific missense and in-frame deletion variants on mTORC1 activity using in vitro functional analysis, demonstrating the importance of specific amino acid residues in TSC2 for TSC1/2 function.

## 2. Materials and Methods

### 2.1. Ethical Considerations

This study was approved by the Institutional Ethics Review Boards of the four participating institutions (main protocols under certificate of presentation for ethical consideration numbers CAAE 12572913.3.0000.5464 and CAAE 48259715.2.0000.5464). All patients had informed consent signed by a parent or tutor to provide a blood sample for DNA extraction and analysis.

### 2.2. Patient Cohort

A definite clinical diagnosis of TSC [1,2] was the only inclusion criterion. Individuals were referred for testing from three clinical centers: 74 were referred from the Clinics Hospital of the Universidade Federal do Paraná (UFPR), Curitiba, Brazil; 23 from the Child Neurology service of São Paulo Santa Casa de Misericórdia (SPSC), São Paulo, Brazil; and 19 from Grupo de Apoio ao Adolescente e Criança com Câncer (GRAACC), São Paulo, Brazil. Genetic counseling was offered to all families.

### 2.3. DNA Analysis

Peripheral blood samples (4 mL) were harvested by venipuncture and sent to the University of São Paulo (Instituto de Biociências, University of São Paulo, São Paulo, Brazil) for molecular testing. Genomic DNA was extracted from peripheral blood leukocytes using the QIASymphony kit (QIAGEN, Germantown, MD, USA) and quantified on a NanoDrop 2000 spectrophotometer (Thermo Fisher Scientific, Waltham, MA, USA). Analysis of subject DNA samples was performed over an extended period (years 2014–2022), following the arrival of the samples to the laboratory. The choice of the DNA sequencing method, either Sanger or next-generation sequencing (NGS), was solely based on accessibility to equipment. All samples that did not have *TSC1* and *TSC2* sequences fully sequenced by the Sanger method were submitted to NGS.

### 2.4. PCR and Sanger Sequencing

Oligonucleotides for PCR and Sanger sequencing were designed with Primer3 v. 0.4.0 [20]. PCR amplicons covered all exons, intron boundaries, and the core promoter regions of both *TSC1* (NG_012386.1) and *TSC2* (NG_005895.1) (Appendix A Table A1 and Table A2). For *TSC1*, the sequenced region consisted of ~9.4 kb of the total genomic locus (60 kb; ~16%), including 517 bp of the core promoter [21] and upstream sequences, 3.5 kb of exonic sequences, and 5.4 kb of intronic sequences. For *TSC2,* the sequenced region consisted of ~17.8 kb (46 kb; ~39%), including 485 bp of the core promoter and upstream sequence, 5.6 kb of exonic sequences, and 11.7 kb of intronic sequences. Coding sequence annotation was according to reference transcripts NM_000368.4 (*TSC1*) and NM_000548.3 (*TSC2*). Primer specificity was tested against the human genome build GRCh37/hg19 with the BLAT program (UCSC Genome Browser, http://genome.ucsc.edu/cgi-bin/hgBlat?command=start, accessed on 13 June 2024) and nucleotide BLAST (BLAST NCBI, http://blast.ncbi.nlm.nih.gov, accessed on 13 June 2024). The conditions for each PCR were standardized with DNA samples from three unrelated non-TSC individuals (non-TSC control). For segments with a high proportion of cytosine and guanine, we adopted the slowdown PCR protocol [22]. PCR products were electrophoresed on 1.5% agarose gels and images were captured on a Gel Doc™ EZ System using Image Lab™ software (Version 6.1; Bio-Rad, Hercules, CA, USA). For Sanger sequencing, PCR products were treated with exonuclease I/shrimp alkaline phosphatase (5U:1U; Affymetrix, Santa Clara, CA, USA) for 60 min at 37 °C, followed by enzymatic inactivation for 15 min at 60 °C, prior to Sanger sequencing according to the ABI BigDye terminator protocol (Applied Biosystems, Thermo Fisher Scientific, Waltham, MA, USA). Reaction products were purified using Sephadex columns (Cytiva Life Sciences, Wilmington, DE, USA) and submitted to capillary electrophoresis on an ABI 3730xl DNA Analyzer (Applied Biosystems, Thermo Fisher Scientific, Waltham, MA, USA). The results were analyzed using Sequencher 5.3 (Gene Codes Corporation, Ann Arbor, MI, USA).

### 2.5. Next-Generation Sequencing (NGS)

For NGS, DNA library preparation and capture of coding and intronic boundary sequences for both *TSC1* and *TSC2* were performed using Nextera rapid capture (Illumina, San Diego, CA, USA) with specific probes designed as part of a custom gene panel. The library was quantified using the Qubit 2.0 fluorometer (Applied Biosystems, Thermo Fisher Scientific, Waltham, MA, USA) and Bioanalyzer 2100 (Agilent Technologies, Santa Clara, CA, USA), and sequencing was performed on the MiSeq platform (Illumina, San Diego, CA, USA), employing a mean read depth of 195.26 (standard deviation [sd]: 34.18). An average >99.3% coverage of target regions at a minimum depth of 10 reads per nucleotide and >98.9% coverage at depth of 20 reads were obtained (Appendix A Table A3). A minimum threshold of 20 reads and a variant allele frequency (VAF) >40% were employed. VUSs, probable pathogenic, or pathogenic variants detected by NGS were confirmed by PCR followed by Sanger sequencing. All reads were aligned to the human genome (build GRCh37/hg19) using the BWA algorithm [23], followed by GATK [24] and ANNOVAR variant calling and annotation [25].

### 2.6. Multiplex Ligation-Dependent Probe Amplification (MLPA)

The SALSA MLPA kits P124-C1 TSC1 and P046-C1 TSC2 (MRC-Holland, Amsterdam, The Netherlands) were used for detection of duplications and deletions affecting *TSC1* and *TSC2*, essentially as described previously [15,26,27]. Three non-TSC control samples were used as reference samples. Ligated products were separated by capillary electrophoresis on an ABI 3730xl DNA Analyzer (Applied Biosystems, Thermo Fisher Scientific, Waltham, MA, USA) and analyzed using Coffalyser.NET software (MRC-Holland, Amsterdam, The Netherlands). Signal intensities were compared per subject using Student’s *t* test (*p* value < 0.05). Peak heights of <0.70 or >1.30 were considered deletion or duplication of a probe. Reductions or increases in peak height resulting in values between 0.70 and 1 or 1 and 1.30, respectively, were considered indicative of mosaicism and confirmed by quantitative PCR [28].

### 2.7. Quantitative PCR

Quantitative PCR (qPCR) was employed to validate MLPA data and to further assess the extension of the segmental deletions and duplication. It was performed as described previously [29] using the SYBR Green system (Applied Biosystems) on a 7500 Fast Real-Time PCR System apparatus (Applied Biosystems). Primers were designed using Primer-Blast (http://www.ncbi.nlm.nih.gov/tools/primer-blast/, accessed on 13 June 2024) and tested in silico with the Beacon Designer Free Edition (http://www.premierbiosoft.com/qOligo/Oligo.jsp?PID=1, accessed on 13 June 2024), Blat-UCSC Genome Browser (GRCh37/hg19 assembly), and BLAST-NCBI (Appendix A Table A4). Optimal DNA and primer concentrations were determined by titration, leading to the following conditions for a 12 µL reaction: 25 ng DNA and 4 pmol of each primer with 6 µL PCR SYBR Green master mix. A non-TSC control sample was used as reference. Amplification parameters were 95 °C for 10 min, followed by 30 cycles at 95 °C for 15 s and 60 °C for 60 s, with fluorescence acquisition at the end of each step. A melting step (dissociation curve) was performed following each run to confirm product specificity and the absence of primer dimers. For copy number calibration, the reaction was normalized to *GADPH* (Glyceraldehyde-3-phosphate dehydrogenase, NM_002046.1, human chromosome 12). All samples were run in triplicate, and the data were analyzed using the comparative ΔΔCt cycle threshold method. A ratio coefficient (RQ) between 0.7 and 1 was considered indicative of mosaic deletion. Student’s *t* test with significance for *p* value < 0.05 was applied to compare test and reference sample results.

### 2.8. In Silico Analysis and Structural Assessment of DNA Variants

Variant nomenclature was noted according to the recommendations of the Human Genome Variation Society (HGVS) and was checked using both the Variant Validator (University of Leicester [30]) and Mutalyzer (LUMC [31]) web tools. Pathogenic DNA variants were defined according to ACMG standards and guidelines [32].

All DNA variants were submitted to the Variant Effect Predictor (VEP) web tool (https://www.ensembl.org/Tools/VEP, accessed on 13 June 2024) and allele frequencies were verified in databases of human population sequence variants (1000 Genomes, gnomAD, ExaC and ABraOM). In addition, variants were evaluated by comparison with the *TSC1* and *TSC2* Leiden Open Variation Databases (LOVD) (Version 2; Leiden, The Netherlands), Ensembl (http://www.ensembl.org/index.html, release 93—July 2018) [33], PolyPhen-2 [34], Mutation Taster [35], SIFT [36,37], PROVEAN [38], PhosphositePlus [39], Alamut Visual (Interactive biosoftware, version 2.7-1). The Combined Annotation Dependent Depletion (CADD—v1.6) was calculated for all SNVs (single nucleotide variants) using the webtool CADD (https://cadd.gs.washington.edu/, accessed on 13 June 2024) [40]. In silico analysis of variants potentially affecting pre-mRNA splicing was performed using Acescan2, SpliceAid2 [41], Human Splicing Finder [42], and Alamut Visual. To identify repetitive elements, RepeatMasker (Version open-3.0, Institute for Systems Biology, Seattle, WA, USA) was used. Final assessment of DNA variants was in June 2024. Information on all identified DNA variants has been deposited in the *TSC1* and *TSC2* LOVD (https://www.lovd.nl/TSC1; https://www.lovd.nl/TSC2) and ABraOM (https://abraom.ib.usp.br/, accessed on 13 June 2024) databases.

Alignment of the *Homo sapiens* TSC2 (NP_000539.2) and *Homo sapiens* RAP1GAP (NP_001337453.1) GAP domains [43] was performed using UniProtKB BLAST (EMBL-EBI, https://www.uniprot.org/blast, accessed on 13 June 2024). Human RAP1GAP crystal structure was retrieved from the protein data bank (PDB accession number 1SRQ; http://www.wwpdb.org, accessed on 13 June 2024), and the variant was highlighted in the space-filling diagram using PyMOL (Graphic System Version 2.1.1; http://www.pymol.org, accessed on 13 June 2024; maintained by Schrödinger, San Diego, CA, USA).

### 2.9. Functional Assessment of TSC2 Variants

Functional assessment was performed as previously described [44,45], except that a HEK293T cell line (3H9-1B1), in which both *TSC1* and *TSC2* genes had been inactivated by CRISPR/Cas9 genome editing, was used [46]. Briefly, a full-length *TSC2* expression construct encoding the variant of interest was derived by site-directed mutagenesis. All constructs were verified by Sanger sequencing. The transfections were performed, and cells were lysed 18 h later. The cleared cell lysates were submitted to SDS-PAGE and transferred to nitrocellulose membranes. All blots were scanned using the Odyssey scanner (Li-Cor Biosciences, Lincoln, NE, USA). Signal intensities were measured and normalized to the intensities corresponding to wild-type *TSC2*, and the ratio of the signal for S6K phosphorylated at Thr389 to the total S6K (T389/S6K) signal was determined for each variant.

## 3. Results

Review of the available clinical data identified 116 individuals, 53 male and 63 female, who fulfilled the clinical criteria for definite TSC [2]. Age at the time of enrolment varied between 5 months and 19 years (mean: 9.62 years; median: 11 years). In total, DNA samples from 116 individuals were assessed by Sanger sequence analysis of polymerase chain reaction (PCR) products for both *TSC1* and *TSC2*. In 40 cases, DNA was also analyzed by MLPA, and DNA from 21 subjects was submitted to next-generation sequencing (NGS), in addition to Sanger sequencing and MLPA.

### 3.1. TSC1 and TSC2 Variant Identification

#### 3.1.1. DNA Sequencing

**Figure 1:**
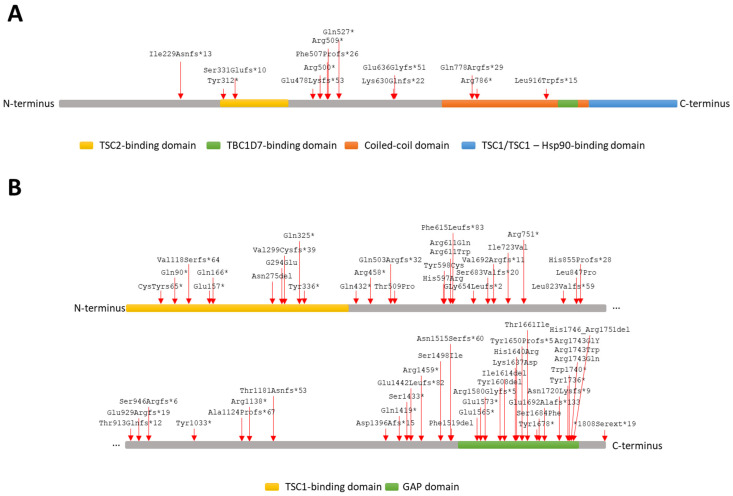
Schematic representation of TSC1 (**A**) and TSC2 (**B**) protein domains according to NP_000359.1 and NP_000539.2. (**A**) Location of each pathogenic variant detected in *TSC1.* (**B**) Location of each pathogenic variant detected in *TSC2.* Splicing variants and CNVs are not represented in this figure.

A total of 262 distinct DNA variants was detected. Of these, 90 were classified as pathogenic according to the ACMG guidelines (Table 1 and Table 2; Figure 1 [32]); 151 were frequent SNVs (population frequency > 0.01); and 21 were novel and not present in 1000 Genomes, ABraOM or dbSNP data banks but were not classified as pathogenic according to the ACMG guidelines. Of these novel variants, 18 (86%) were identified in subjects with a pathogenic *TSC1* or *TSC2* variant (Appendix A Table A5 and Table A6).

In total, seven recurrent pathogenic variants, three in *TSC1* and four in *TSC2,* were identified in 15 unrelated individuals. The remaining pathogenic variants were identified in singleton individuals. To our knowledge, 6 pathogenic *TSC1* and 28 pathogenic *TSC2* variants have not been reported previously (Table 1 and Table 2).

**Figure 2:**
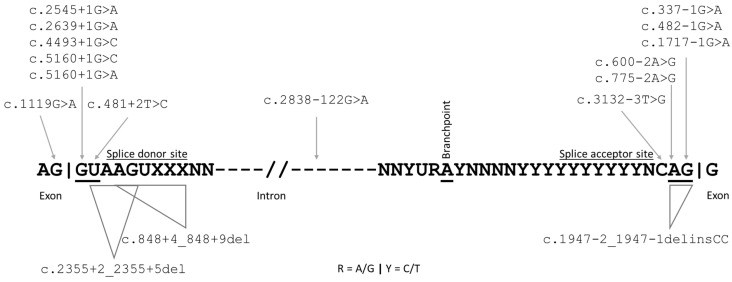
Schematic representation of pathogenic *TSC2* splice variants. Intron canonical splice sites and splicing branchpoint are underlined in a putative RNA sequence. Exon-intron borders are represented by vertical bars.

Splicing variants were only identified in *TSC2*: 13 variants disrupted a canonical splice donor or acceptor site, and 5 variants were either exonic, deep intronic, or adjacent to the acceptor site (Table 2 and Figure 2). One variant, c.2838-122G>A, had been reported previously as pathogenic in multiple TSC cases [17,47]. The rare synonymous variant, c.1119G>A, affecting the last nucleotide of *TSC2* exon 11, was considered pathogenic because it leads to exon 11 skipping, as recently reported by analysis of leukocyte cDNA of a TSC patient [48]. Skipping exon 11 causes an in-frame deletion in the TSC2 hamartin-binding domain (p.(A326_Q373del)). The variant c.3132-3T>G is predicted to cause skipping of exon 28, leading to an in-frame deletion of 50 amino acids (TSC2 p.R1044_S1094del). In silico analysis of variant c.848+4_848+9del predicted skipping of exon 8, leading to a frameshift: p.L259Sfs*52. Finally, although classified as a missense change (Table 2), the c.4493G>T, p.(S1498I) variant affects the last nucleotide of exon 34 and is predicted to result in skipping of exon 34: p.S1336fs*25.

#### 3.1.2. MLPA Analysis for Copy Number Variation Identification in *TSC1* and *TSC2*

MLPA was performed on DNA samples from 40 individuals, including 20 who remained NMI after PCR/Sanger sequence analysis. We identified one deletion encompassing *TSC1* exons 9 through 23 (*TSC1*:c.(737+1_738-1)_(*1+_?)del); Table 1), six deletions partially or totally encompassing *TSC2,* and a duplication of *TSC2* (Table 2 and Table 3).

The *TSC1*:c.(737+1_738-1)_(*1+_?)del (PS = 0.55, *p*-value = 1.06 × 10^−8^) was corroborated by qPCR (RQ = 0.41; SEM = 0.01; *p*-value = 7 × 10^−4^) and found to extend into the adjacent *SPACA9* locus (qPCR of *SPACA9* exon 4: RQ = 0.34; SEM = 0.06; *p* = 9.3 × 10^−3^).

**Figure 3:**
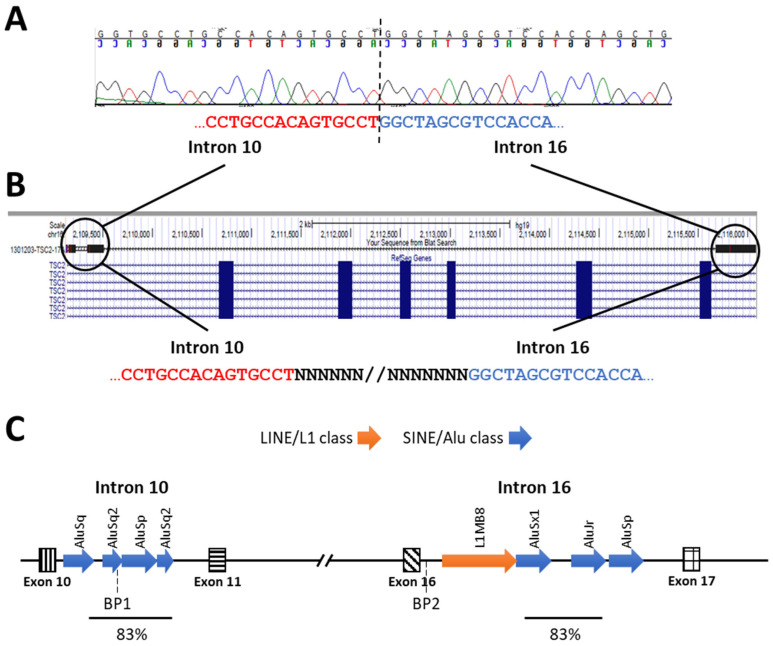
*TSC2* c.(975+627)_(1716+41)del intragenic deletion. (**A**) Sanger sequencing of the intragenic *TSC2* deletion c.(975+627)_(1716+41)del identifies the breakpoints. (**B**) UCSC genome browser mapping of the breakpoints in *TSC2* introns 10 and 16. (**C**) Diagram illustrating the positions of SINE/Alu and LINE/L1 repeat sequences in *TSC2* introns 10 and 16, respectively, within and adjacent to each breakpoint (BP1 and BP2). Exons are shown as patterned boxes. The segments of introns 10 and 16 that display 83% similarity are indicated by lines.

The six *TSC2* deletions and duplication detected by MLPA were confirmed by qPCR (Table 3). Analysis of the 6.2 kb intragenic *TSC2* deletion c.975+627_1716+41del was indicative of somatic mosaicism by MLPA (PS = 0.74, *p*-value = 1.13 × 10^−4^), although unconfirmed by qPCR (Table 3). PCR and Sanger sequencing identified the breakpoints in *TSC2* introns 10 and 16, close to multiple repetitive elements, two of them with high homology (Figure 3).

Two deletions and the duplication extended to the 3′ end of the *TSC2* gene. To evaluate whether these rearrangements affected *PKD1*, exons 45 (NG_008617.1:g.50379-50490 targeted region) and 46 (NG_008617.1:g.51726–51832) of *PKD1* were tested by qPCR. *TSC2* deletion (c.(5068+1_5069-1)_(*102_?)del) was consistent with somatic mosaicism (Table 3) and extended from *TSC2* exon 39 through *PKD1* exon 46, as validated by qPCR (RQ = 0.57; SEM = 0.06; *p* = 0.02); exon 45 was preserved (RQ = 1.0; SEM = 0.2; *p* = 0.99). Similarly, the *TSC2* c.(?_-30)_(*102_?)del deletion encompassed *PKD1* exon 46 (RQ = 0.58; SEM = 0.02; *p* = 2.2 × 10^−3^) but not exon 45 (RQ = 0.92; SEM = 0.1; *p* = 0.42). In addition, *NTHL1* exon 6, upstream of *TSC2,* was deleted (RQ = 0.50; SEM = 0.05; *p* = 8.6 × 10^−3^).

*PKD1* exons 45 and 46 of the individual with the segmental duplication were not different from controls, as assessed by qPCR (exon 45: RQ = 1.18, SEM = 0.1; *p* = 0.14; exon 46: RQ = 0.83; SEM = 0.09; *p* = 0.19). We could not confirm if the *TSC2* partial duplication was in tandem.

### 3.2. Functional Analyses of TSC2 Missense and In-Frame Deletion Variants

We performed in vitro functional testing of ten *TSC2* variants: p.L18V, p.W167R, p.T509P, p.I723V, p.L847P, p.R1044_S1094del, p.S1498I, p.Y1608del, p.I1614del, and p.R1743G. The variant p.R611Q (c.1832G>A), previously shown to be pathogenic [49,50], was employed as a positive control.

**Figure 4:**
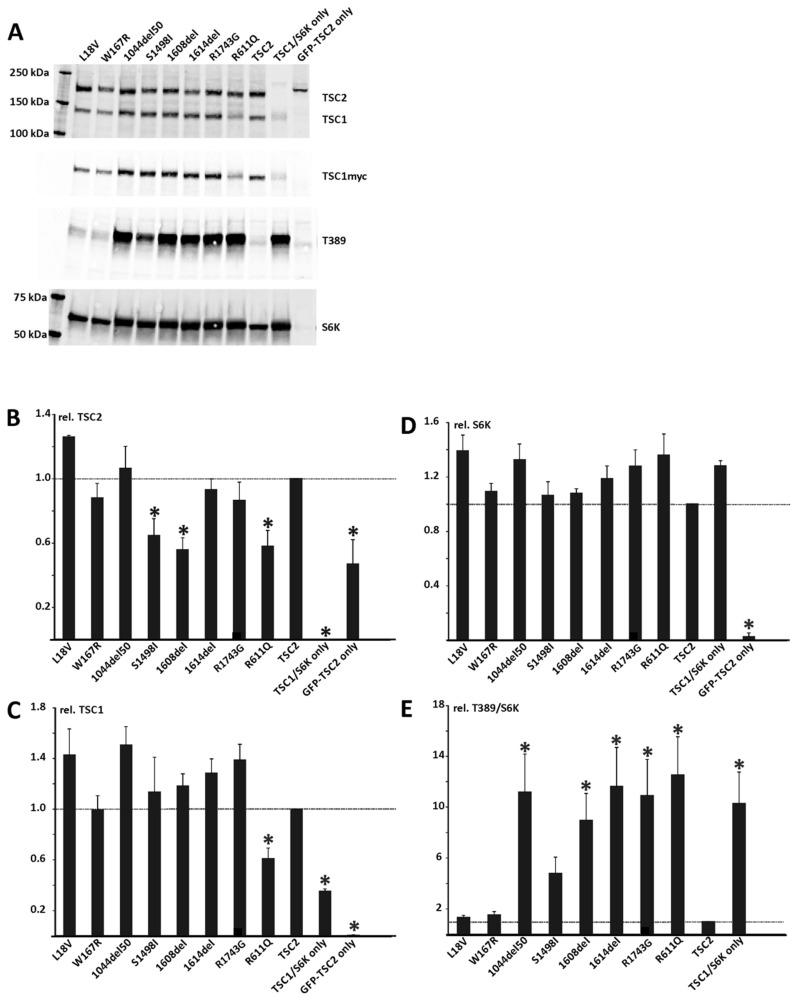
Functional assessment of the *TSC2* c.52C>G (p.L18V), c.499T>C (p.W167R), c.3132_3282del (p.1044_1094del) indicated as 1044del50, c.4493G>T (p.S1498I), c.4823_4825del (p.1608del), c.4840_4842del (p.1614del), and c.5227C>G (p.R1743G) variants. 3H9-1B1 (*TSC2*/*TSC1* double knockout HEK 293T) cells were transfected with the indicated *TSC2* variant expression constructs, together with expression constructs for myc-tagged TSC1 and S6K. WT-TSC2 as well as the pathogenic *TSC2* c.1832G>A (p.R611Q) variant (R611Q) and cells with no *TSC2* expression (TSC1/S6K only) were included as controls. GFP-TSC2 (GFP-TSC2 only) was employed to monitor transfection efficiency. Twenty-four hours after transfection, the cells were harvested, and the cleared cell lysates were analyzed by immunoblotting (**A**). The signals for TSC2, TSC1, total S6K (S6K), and T389-phosphorylated S6K (T389) were determined per variant, relative to the wild-type control (TSC2) in two independent experiments. Mean TSC2 (**B**), TSC1 (**C**), and S6K (**D**) signals and mean T389/S6K ratio (**E**) are shown for each variant. In each case, the dotted line indicates the signal/ratio for wild-type TSC2 (normalized to 1.0). Error bars represent the standard error of the mean. Statistical significance for comparisons with WT-TSC2 is indicated with an asterisk (*p* < 0.025; Student’s paired *t*-test).

**Figure 5:**
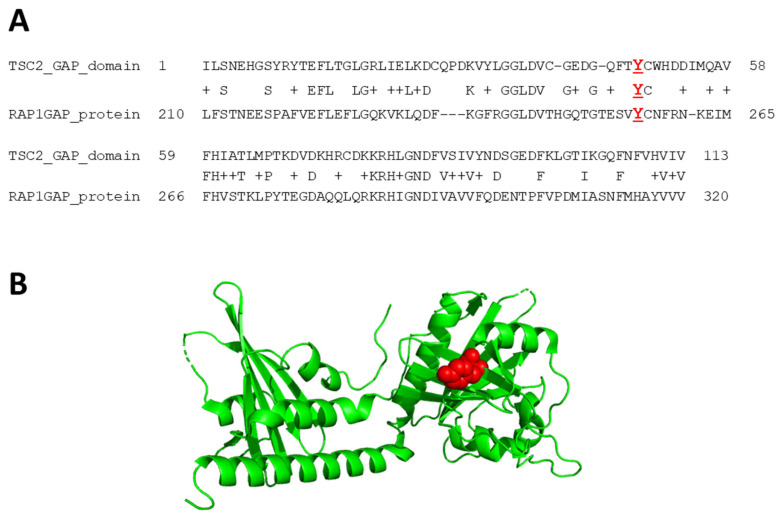
Structural analysis of TSC2 variants affecting the TSC2 GAP domain. (**A**) Amino acid sequence alignment of human TSC2 (NP_000539.2) and the human RAP1GAP domain (NP_001337453.1). TSC2 Y1608 and RAP1GAP Y256 are indicated in red. (**B**) The TSC2:Y1608 residue is highlighted in red in the gray β-sheet part of the ribbon diagram of protein data bank structure (accession number: 1SRQ).

The p.S1498I and p.1608del variants resulted in significantly decreased TSC2 signals (Figure 4A,B), and the p.T509P, p.L847P, and p.R611Q variants were associated with reduced TSC1 and TSC2 signals (Figure 4A–C). The p.T509P, p.L847P, p.R1743G, p.Y1608del, p.I1614del, and p.R1044_S1094del variants were unable to inactivate TORC1-dependent S6K-T389 phosphorylation, similar to the pathogenic p.R611Q variant (Figure 4A,E). The Y1608 residue within the TSC2 GAP domain is conserved among *TSC2* orthologues and human RAP1 GAP (residue Y256; Figure 5).

In contrast, the p.L18V, p.W167R, and p.I723V variants did not disrupt TSC1/2 function in our assay: S6K-T389 phosphorylation (T389/S6K ratio) was not significantly different to wild-type (WT) TSC2 (Figure 4E). The p.S1498I variant exhibited an intermediate effect on TSC1/2 function. The T389/S6K ratio for the p.S1498I variant was significantly reduced compared to the inactive p.R611Q variant (*p* = 0.002; paired Student’s *t*-test, with Bonferroni correction) but was still increased more than three-fold relative to WT TSC2 (*p* = 0.042) (Figure 4).

Altogether, among the 116 unrelated individuals clinically diagnosed with TSC, 18 had pathogenic alterations in the *TSC1* gene and 88 in *TSC2*, yielding a mutation detection rate of 91.4% (106/116; Table 1, Table 2 and Table 4). Finally, the *TSC2* variant c.1443G>T (p.E481D) that does not inactivate the TSC1/2 (Nellist, personal communication) was identified in an NMI patient. The c.1443G>T substitution is predicted to disrupt the 5′ donor site of exon 14, possibly resulting in skipping of this exon: r.1362_1443del, (p.R454fs*3). The same variant has been identified in two unrelated individuals with TSC from other studies, but in the absence of RNA data, we considered the variant a VUS.

## 4. Discussion

As a multisystem disorder owing to loss of function of the tumor suppressor genes *TSC1* or *TSC2*, TSC has variable expressivity. TSC morbidity commonly relates to refractory epilepsy, kidney angiomyolipoma leading to acute bleeding and/or renal insufficiency, and pulmonary lymphangioleiomyomatosis (LAM). Proper control of epilepsy in the first two years of life is directly associated with better cognitive outcome. In addition, untreated growing subependymal giant cell astrocytoma (SEGA) may aggravate epilepsy and lead to hydrocephalus. The availability of mTOR inhibitors for clinical treatment of TSC patients with growing angiomyolipoma, SEGA, LAM, and as adjuvant therapy for refractory epilepsy positively impacts the clinical outcome and highlights the need for early diagnosis of the disease [2].

Molecular analysis of *TSC1* and *TSC2* was performed in this study in 116 Brazilian individuals with a definite clinical diagnosis of TSC. Individuals were referred from three large tertiary clinics. We report a mutation detection rate (106/116; 91.4%) similar to previous reports but with a relatively high ratio (1:5) of cases with an inactivating variant in *TSC2* [5,26,51,52,53,54,55,56]. The three referring hospitals are from two neighboring states, São Paulo and Paraná, respectively, in the southeast and south of Brazil. A Brazilian report on TSC patients from these two regions disclosed a *TSC1*:*TSC2* pathogenic alteration ratio of 1:2.5 [57]. In our study, two clinics that together contributed 97 TSC patient DNA samples are referral centers for epilepsy treatment, which might lead to a higher recruitment of severe, refractory epilepsy cases and therefore a higher rate of mutation detection as well as a preponderance of pathogenic *TSC2* variants [12,51,52]. The third hospital is a reference for brain tumor treatment and follows up severe patients with genetic tumor syndromes referred to by neurologists, which may additionally justify a higher number of patients with *TSC2* alterations. 

Although this study did not aim at clinical characterization, it is possible that case severity might explain the limited number of NMI cases or the high frequency of *TSC2* pathogenic variants. Contiguous gene deletion syndrome, characterized by both TSC and polycystic kidney disease, affects up to 5% of TSC patients [2] and has been diagnosed in the two patients with *TSC2* segmental deletions encompassing *PKD1* exon 46, assisted in a multispecialty clinic. The largest cohort of patients from an individual hospital in this study (*n* = 74) disclosed a *TSC1*:*TSC2* mutation ratio of 1:8.9 (7:62) and is mostly composed of sporadic cases, according to family reports. The low number of patients with a *TSC1* pathogenic alteration in this cohort prevented a robust genotype–phenotype correlation as recently reported [58,59]. The cohort from another epilepsy center (*n* = 23) has a *TSC1*:*TSC2* ratio of 1:1.4 (9:13) and more familial cases. This is consistent with previous reports of a significantly higher number of *TSC2* pathogenic alterations among sporadic TSC cases [51,52]. Moreover, NMI cases appear milder and frequently consist of TSC patients whose pathogenic variant presents somatic mosaicism or lies in regulatory segments of either *TSC1* or *TSC2* genes [13].

We identified novel DNA variants, including 35 pathogenic alterations (Table 1 and Table 2, Appendix A Table A5 and Table A6). Nonsense and frameshifting variants were the most common types of pathogenic DNA variants for both genes (Table 3). Splicing variants constituted an important fraction of *TSC2* pathogenic alterations (Table 2) and VUSs (Appendix A Table A6).

Well-established in vitro functional studies supportive of a damaging effect on a gene product or pathway have been classified as a strong criterion for helping determine the pathogenicity of missense variants [32]. Functional assessment of *TSC1* and *TSC2* missense variants can provide insight into their effects on mTORC1 activity [44,45,60]. We investigated seven missense variants and three predicted in-frame deletions affecting *TSC2*. Four missense variants, two in-frame deletions, and the predicted in-frame 50-amino acid deletion (p.1044del50) inactivated the TSC1/2 complex and were thus classified as pathogenic. The four missense variants, p.T509P, p.L847P, p.S1498I, and p.R1743G, clearly disrupted TSC1/2 complex activity in our functional assay. Although the c.4493G>T (p.S1498I) did not completely inactivate the TSC1/2, we considered the clear disruption of activity as good evidence to support pathogenicity. Furthermore, the c.4493G>T variant is predicted to disrupt *TSC2* pre-mRNA splicing.

We classified the p.L18V, p.W167R, and p.I1723V variants as unlikely to be pathogenic. These variants did not have a significant effect on TSC1/2 activity in our assay and, in each case, other pathogenic changes were identified in the individual concerned (Table 5).

In our study, the c.1443G>T (p.Glu481Asp) variant was detected in two individuals. In one of these cases, we also identified the *TSC2* c.1790A>G (p.H597R) pathogenic variant. Functional testing and RNA studies should help resolve whether these variants are likely to be pathogenic.

Among seven large deletions (7/106; 6.6%), two were analyzed in detail, one extending from *TSC1* c.(737+1_738-1)_(*1+_?)del) into the downstream *SPACA9* locus and another mapping into *TSC2* c.(975+628)_(1716+41)del. In this case, the breakpoints were identified and shown to lie close to Alu repeats (Figure 4). The high identity between the Alu-Sp repeat in *TSC2* intron 10 and the Alu-Sq repeat in intron 16 suggests homologous unequal recombination or intra-chromosomal recombination as likely mechanisms to generate the deletion [61,62,63].

In summary, 106 out of 116 individuals with a clinical diagnosis of TSC had pathogenic DNA alterations in *TSC1* or *TSC2* identified by DNA sequencing or MLPA, confirmed by functional assessment or qPCR, if indicated. We detected 35 novel pathogenic DNA alterations as well as Alu-associated breakpoints leading to an intergenic *TSC2* deletion. Functional assessment of *TSC2* missense and in-frame deletion variants increased the number of variants that could be classified as (likely) pathogenic by ~7% and identified novel residues in the TSC2 protein that are critical for TSC1/2 complex function.

## Figures and Tables

**Table 1:** *TSC1* pathogenic DNA variants in a cohort of 116 TSC patients.

DNA Variant Type	DNA Variant	Location	Reported ^e^	Frequency ^f^	CADD ^g^
Frameshift	c.683dup (p.I229Nfs*13) ^a^	Exon 08	-	-	-
Frameshift	c.989dup (p.S331Efs*10)	Exon 10	30	-	-
Frameshift	c.1431_1434del (p.E478Kfs*53) ^d^	Exon 14	14	-	-
Frameshift	c.1517_1518dup (p.F507Pfs*26) ^a^	Exon 15	-	-	-
Frameshift	c.1888_1891del (p.K630Qfs*22)	Exon 15	58	-	-
Frameshift	c.1907_1908del (p.E636Gfs*51)	Exon 15	16	6.19 × 10^−7^	-
Frameshift	c.2332del (p.Q778Rfs*29) ^a^	Exon 18	-	-	-
Frameshift	c.2746del (p.L916Wfs*15) ^a^	Exon 21	-	-	-
Nonsense	c.936C>G (p.Y312*) ^a^	Exon 10	-	-	34
Nonsense	c.1498C>T (p.R500*) ^d^	Exon 15	44	-	39
Nonsense	c.1525C>T (p.R509*) ^d^	Exon 15	73	-	38
Nonsense	c.1579C>T (p.Q527*)	Exon 16	7	-	36
Nonsense	c.2356C>T (p.R786*)	Exon 18	48	-	41
Nonsense	c.2626G>T (p.E876*) ^a^	Exon 21	-	1.57 × 10^−6^	47
Large deletion	c.(737+_738-)_(*1+_?)del ^b,c^	Exons 9–23	-	-	-

^a^ First description of this mutation. ^b^ NGS and MLPA performed for both genes, in addition to Sanger sequencing. ^c^ Deletion confirmed by qPCR. ^d^ Same mutation in two unrelated patients. ^e^ Total reported cases according to LOVD-*TSC1* (June/2024). ^f^ Frequency according to gnomAD data bank (June/2024). ^g^ CADD (Combined Annotation Dependent Depletion) v1.6.

**Table 2:** *TSC2* pathogenic DNA variants in a cohort of 116 TSC patients.

	DNA Variant Type	DNA Variant		Functional Testing *	Location	Reported ^g^	Frequency ^n^	CADD ^o^
1.	Frameshift	c.352del (p.V118Sfs*64) ^a^		NR	Exon 05	-	-	-
2.	Frameshift	c.894dup (p.V299Cfs*39)		No	Exon 10	5	-	-
3.	Frameshift	c.1507del (p.Q503Rfs*32) ^a^		NR	Exon 15	-	-	-
4.	Frameshift	c.1842del (p.F615Lfs*83)		NR	Exon 18	-	-	-
5.	Frameshift	c.1959_1960del (p.G654Lfs*2)		No	Exon 19	5	-	-
6.	Frameshift	c.2046dup (p.S683Vfs*20)		No	Exon 19	2	-	-
7.	Frameshift	c.2073dup (p.V692Rfs*11) ^a^		NR	Exon 19	-	-	-
8.	Frameshift	c.2467_2476delinsGTGGATGA (p.L823Vfs*59) ^a^		NR	Exon 21	-	-	-
9.	Frameshift	c.2563dup (p.H855Pfs*28) ^a^		NR	Exon 23	-	-	-
10.	Frameshift	c.2737_2739delinsC (p.T913Qfs*12) ^a^		NR	Exon 24	-	-	-
11.	Frameshift	c.2784del (p.E929Rfs*19)		No	Exon 25	2	-	-
12.	Frameshift	c.3370del (p.A1124Pfs*67) ^a^		NR	Exon 29	-	-	-
13.	Frameshift	c.3541dup (p.T1181Nfs*53) ^a^		NR	Exon 30	-	-	-
14.	Frameshift	c.4187del (p.D1396Afs*15) ^a^		NR	Exon 34	-	-	-
15.	Frameshift	c.4324_4327delinsCTTCT (p.E1442Lfs*82) ^a^		NR	Exon 34	-	-	-
16.	Frameshift	c.4544_4547del (p.N1515Sfs*60)		No	Exon 35	31	-	-
17.	Frameshift	c.4738del (p.R1580Gfs*5) ^a^		NR	Exon 37	-	-	-
18.	Frameshift	c.4947_4948insCCATTGT (p.Y1650Pfs*5) ^a^		NR	Exon 38	-	-	-
19.	Frameshift	c.5075_5078del (p.E1692Afs*133) ^a^		No	Exon 40	3	-	-
20.	Frameshift	c.5159dup (p.N1720Kfs*9) ^a^		NR	Exon 40	-	-	-
21.	Nonsense	c.195T>A (p.C65*) ^a^		NR	Exon 03	-	-	34
22.	Nonsense	c.268C>T (p.Q90*) ^h^		No	Exon 04	32	-	36
23.	Nonsense	c.496C>T (p.Q166*) ^i^		No	Exon 05	7	-	41
24.	Nonsense	c.973C>T (p.Q325*)		No	Exon 10	4	-	36
25.	Nonsense	c.1008T>G (p.Y336*)		No	Exon 11	3	-	32
26.	Nonsense	c.1195G>T (p.E399*)		No	Exon 12	2	-	38
27.	Nonsense	c.1294C>T (p.Q432*)		No	Exon 13	3	-	37
28.	Nonsense	c.1372C>T (p.R458*)		No	Exon 14	50	-	38
29.	Nonsense	c.2251C>T (p.R751*)		No	Exon 21	46	-	38
30.	Nonsense	c.3099C>G (p.Y1033*)		No	Exon 27	10	-	33
31.	Nonsense	c.3412C>T (p.R1138*)		No	Exon 30	62	-	46
32.	Nonsense	c.4255C>T (p.Q1419*)		No	Exon 34	15	-	47
33.	Nonsense	c.4298C>A (p.S1433*)		No	Exon 34	2	-	36
34.	Nonsense	c.4375C>T (p.R1459*)		No	Exon 34	49	6.23 × 10^−8^	40
35.	Nonsense	c.4693G>T (p.E1565*)		No	Exon 37	1	-	49
36.	Nonsense	c.4716_4717delGGinTT (p.E1573*) ^a^		NR	Exon 37	-	-	-
37.	Nonsense	c.5034C>A (p.Y1678*)		No	Exon 39	2	-	27.4
38.	Nonsense	c.5208C>G (p.Y1736*) ^a^		NR	Exon 41	-	-	37
39.	Nonsense	c.5220G>A (p.W1740*)		No	Exon 41	4	-	54
40.	Missense	c.1525A>C (p.T509P) ^k^		Yes	Exon 15	3		25.9
41.	Missense	c.1663G>C (p.A555P) ^j,k^		Yes	Exon 16	3	3.09 × 10^−6^	22.7
42.	Missense	c.1790A>G (p.H597R)^k^		Yes	Exon 17	9	-	23.6
43.	Missense	c.1793A>G (p.Y598C) ^k^		Yes	Exon 17	7	-	24.6
44.	Missense	c.1831C>T (p.R611W) ^k^		Yes	Exon 17	54	-	27.9
45.	Missense	c.1832G>A (p.R611Q) ^d,k^		Yes	Exon 17	101	-	28.8
46.	Missense	c.2540T>C (p.L847P) ^k^		Yes	Exon 22	8	-	29.4
47.	Missense	c.4493G>T (p.S1498I) ^d^		Yes	Exon 34	2	-	34
48.	Missense	c.4909_4911delinsGAC (p.K1637D) ^l^		Yes	Exon 38	2	-	-
49.	Missense	c.4919A>G (p.H1640R) ^l^		Yes	Exon 38	4	-	25.6
50.	Missense	c.5024C>T (p.P1675L) ^h,l^		Yes	Exon 39	63	-	28.4
51.	Missense	c.5227C>G (p.R1743G) ^d,l^		Yes	Exon 41	4	-	23.3
52.	Missense	c.5227C>T (p.R1743W) ^l^		Yes	Exon 41	57	-	25.2
53.	Missense	c.5228G>C (p.R1743P) ^l^		Yes	Exon 41	10	-	29.9
54.	Missense	c.5228G>A (p.R1743Q) ^l^		Yes	Exon 41	62	-	32
55.	In-frame deletion	c.824_826del (p.N275del) ^k^		Yes	Exon 09	6	-	-
56.	In-frame deletion	c.4823_4825del (p.Y1608del) ^j,l^		Yes	Exon 37	15	-	-
57.	In-frame deletion	c.4842_4844del (p.I1614del) ^j,l^		Yes	Exon 37	22	-	-
58.	In-frame deletion	c.5238_5255del (p.H1746_R1751del) ^l^		Yes	Exon 41	125	6.02 × 10^−6^	-
59.	Large Deletion	c.(774+1_775-1)_(848+1_849-1)del ^a^		No	Exon 09	-	-	-
60.	Large Deletion	c.(975+628)_(1716+41)del ^a,b,c^		No	Exon 11–16	-	-	-
61.	Large Deletion	c.(1599+1_1600-1)_(2545+1_2546-1)del ^a^		No	Exon 16–22	-	-	-
62.	Large Deletion	c.(1716+1_1717-1)_(2545+1_2546-1)del ^a^		No	Exon 17–22	-	-	-
63.	Large Deletion	c.(5068+1_5069-1)_(*102_?)del		No	Exon 39-PKD1	1	-	-
64.	Large Deletion	c.(?_-30)_(*102_?)del ^a^		No	*TSC2*	2	-	-
65.	Large Duplication	c.(2355+1_2356-1)_(*102_?)dup		No	Exon 22–42	1	-	-
66.	Complex	c.5423G>C (p.*1808Sext*19) ^c^		No	Exon 42	2	-	11.8
	DNA variant type	DNA Variant	Predicted effects: [r.spl?] ^e^ or [r.(spl?)] ^f^					
67.	Splicing	c.337-1G>A	[r.spl?]: exon 4 skipping, p.G113Lfs*20	No	Intron 04	2	-	35
68.	Splicing	c.481+2T>C ^a^	[r.spl?]: exon 4 skipping, p.G113Lfs*20	NR	Intron 05	-	-	31
69.	Splicing	c.482-1G>A ^a^	[r.spl?]: exon 6 skipping, p.A161Vfs*22	NR	Intron 05	-	-	33
70.	Splicing	c.600-2A>G	[r.spl?] exon 7 skipping, p.Q200fs*3	No	Intron 06	11	-	26.2
71.	Splicing	c.775-2A>G ^a^	[r.spl?]: exon 9 skipping, p.L259*	NR	Intron 08	-	-	33
72.	Splicing	c.848+4_848+9del ^a^	[r.(spl?)]: exon 8 skipping, p.L259Sfs*52	NR	Intron 09	-	-	-
73.	Splicing	c.1119G>A (p.=)	[r.spl?]: exon 11 skipping, p.Ala326_Gln373del	Yes	Exon 11	4	-	19.54
74.	Splicing	c.1717-1G>A	[r.spl?]: exon 17 skipping, p.T573Sfs*5	No	Intron 16	2	-	34
75.	Splicing	c.1947-2_ 1947-1delinsCC ^a^	[r.spl?]: exon 19 skipping, p.M649fs*7	NR	Intron 18	-	-	-
76.	Splicing	c.2355+2_2355+5del	r.[=,2166_2583ins2355+5_2355+7, 2166_2583ins(2355+1_2356-1)58]	Yes	Intron 21	17	-	-
77.	Splicing	c.2545+1G>A	[r.spl?] exon 21 skipping, p.L741_Q785del	No	Intron 22	5	-	35
78.	Splicing	c.2639+1G>A	[r.spl?] exon 22 skipping, p.R786Lfs45*	No	Intron 23	3	-	34
79.	Splicing	c.2838-122G>A ^h^	[r.(2837_2838ins2838-120_2838-1)], p.S946Rfs*6	Yes	Intron 25	11	-	36
80.	Splicing	c.3132-3T>G ^a,j^	[r.(spl?)]: in-frame exon 28 skipping, p.1044del50	Yes ^m^	Intron 27	-	-	15.64
81.	Splicing	c.4493+1G>C ^a^	[r.spl?]: exon 33 skipping, p.D1295Vfs*77	NR	Intron 34	-	-	33
82.	Splicing	c.5160+1G>C	[r.spl?]: exon 39 skipping, p.D1690Dfs*6	No	Intron 40	3	-	33
83.	Splicing	c.5160+1G>A	[r.spl?]: exon 39 skipping, p.D1690Dfs*6	No	Intron 40	10	-	32

* Some variants have been functionally tested in this study or before according to LOVD TSC2 database. NR: Not reported. ^a^ First description of this mutation. ^b^ NGS and MLPA performed for both genes, in addition to Sanger sequencing. ^c^ Deletion confirmed by qPCR. ^d^ MLPA performed for both genes, in addition to Sanger sequencing. ^e^ [r.spl?]: RNA was not analyzed but the change is expected to affect splicing. ^f^ [r.(spl?)]: RNA was not analyzed but the change might affect splicing. ^g^ Number of references according to LOVD-*TSC2* (April/2024). ^h^ Same mutation in two unrelated patients. ^i^ Same mutation in three unrelated patients. ^j^ Functional assay reported in the present study. ^k^ Amino acid effect on harmatin-binding domain. ^l^ Amino acid effect on GAP domain. ^m^ First description of variant and functional analysis on this study. ^n^ Frequency according to gnomAD data bank (June/2024). ^o^ CADD (Combined Annotation Dependent Depletion) v1.6.

**Table 3:** MLPA and qPCR results for *TSC2* segmental deletions and duplication.

MLPA			qPCR		
Segmental Deletion or Duplication	Exon	PS (*p*-Value)	Exon	RQ (SEM)	*p*-Value
c.(774+1_775-1)_(848+1_849-1)del	09	0.58 (<0.01)	9	0.45 (0.03)	<0.01
c.(975+628)_(1716+41)del *	11–16	0.74 (<0.01)	12	0.62 (0.07)	0.03
c.(1599+1_1600-1)_(2545+1_2546-1)del	16–22	0.56 (<0.01)	19	0.54 (0.01)	<0.01
c.(1716+1_1717-1)_(2545+1_2546-1)del	17–22	0.56 (<0.01)	19	0.63 (0.02)	<0.01
c.(5068+1_5069-1)_(*102_?)del *	39-*PKD1*	0.68 (<0.01)	41	0.75 (0.02)	<0.01
c.(?_-30)_(*102_?)del	01–42	0.54 (<0.01)	12	0.5 (0.03)	<0.01
c.(2355+1_2356-1)_(*102_?)dup	22–42	1.47 (<0.01)	41	2.43 (0.07)	<0.01

MLPA exon refers to the extension of the detected deletion or duplication, while qPCR exon refers to the *TSC2* exon tested to validate MLPA. PS: mean probe signal; *p*-Value: Student’s *t*-test *p*-value; RQ: ratio coefficient; SEM: standard error of mean. * Indicative of somatic mosaicism.

**Table 4:** *TSC1* and *TSC2* pathogenic DNA alterations according to mutation type.

Variant Type	*TSC1*/*TSC2*	Total
Nonsense	8/22	30
Frameshift	9/20	29
Splicing	0/18	18
Missense	0/16	16
Large deletion	1/6	7
In-frame deletion	0/4	4
Large duplication	0/1	1
Complex	0/1	1
Total	18/88	106 (91.4%)
*TSC1*:*TSC2* ratio	1:4.9	
No mutation identified (NMI)	10 (8.6%)	
Total	116	

**Table 5:** Variants with no significant effect on TSC1/2 activity identified in individual with pathogenic variants.

Benign Variant	Pathogenic Variant
c.52C>G (p.L18V)	c.4493G>T (p.Ser1498Ile)
c.499T>C (p.W167R)	c.4375C>T (p.Arg1459*)
c.2167A>G (p.I723V)	c.1195G>T (p.E399*)

## Data Availability

DNA variants identified in the study are openly available in *TSC1* and *TSC2* LOVD (https://www.lovd.nl/TSC1; https://www.lovd.nl/TSC2, after 1 August 2024) and ABraOM (https://abraom.ib.usp.br/, after 1 October 2024) databases.

## Appendix A

**Table A1:** PCR and Sanger sequencing primers for the *TSC1* gene.

Targeted Segment	Name	Oligonucleotide Sequence (5′–3′)
Promoter + exon 01	gTSC1_1S	AAATGTTTAGCCCAGGAAGGA
	gTSC1_1A	GCCGGAGATAGCGTGTAATAA
	gTSC1_2A	CATCTTGGACGTACAGCACCT
	gTSC1_2S	CCGTCTATCCTTCCTTTCGAG
Exon 02	gTSC1_3S	TTGGATTTTAACCCGGAACTC
	gTSC1_3A	TCAGGCACTGAATACAAGCAA
Exon 03	gTSC1_4A	GGGGTTCACTGCATGATTCT
	gTSC1_4S	CCTCTTCATAAACTCGCCAAAG
Exon 04	gTSC1_5S	CAGAACTGTAATGCTGCACAAA
	gTSC1_5A	TTCAAGAATCATGGGTCCTACA
Exon 05	gTSC1_6S	TTTTATCTGCATGACCCTTGC
	gTSC1_6A	CCATACTTGCATGGACAAGGT
Exon 06	gTSC1_7S	CAGTAGAGTTGGGGCTCAGTG
	gTSC1_7A	GCACCCAAGATATTCCCTCA
Exons 07 + 08	gTSC1_8S	CTGAAGAGGAGGGCAGAAGTT
	gTSC1_8A	ATTAGTCCTCCGCCTGTGAA
	gTSC1_9A	AATTTCCCTGTCTGCCGTTA
	gTSC1_29S	CAATCCCTAGGCAGCCACTA
Exon 09	gTSC1_10S	TTTCCATTTTGAGGCTACACC
	gTSC1_10A	TTCCAGAGACAAAGTTGCAAAA
Exon 10	gTSC1_28S	ACCTAAAACCACACACTAACCC
	gTSC1_11A	GGAATGCTAAGTCATCCACGA
Exons 11 + 12	gTSC1_12S	GGGAAAATTTCACACTGCTCA
	gTSC1_12A	CACACCTTGAGAGCAGCTTGT
	gTSC1_13A	CCCAGGGATTTGCAATAAGT
	gTSC1_13S	CGGCAGTTTTTCTAATAGTTGG
Exons 13 + 14	gTSC1_14S	CATCCCAACAATTTGAGAATCA
	gTSC1_14A	GGCATCACTTTACCTGGCATA
	gTSC1_15A	TCCCAGAATTTCCTTGTTTCC
	gTSC1_15S	CCATGTCCAGCCTTCTCTGT
Exon 15	gTSC1_16S	GGATGCCACTTTTTCTCCTCT
	gTSC1_16A	TCCCAATTTAGGTGCACAGAG
	gTSC1_17A	GATGACAAAATGATGGGCTGT
	gTSC1_17S	CACACCAAAGCAAGCCTTTAC
Exons 16+17	gTSC1_18S	TTATGCCATTGCAGATTTTGAC
	gTSC1_18A	GGAAGGACTGGGAACTCTGAC
	gTSC1_19A	ACTTGGCAACACTTGAGATCCT
	gTSC1_19S	AAGCTAACAACACATGGGAAGG
Exon 18	gTSC1_20S	GCAAACTGATCCCTGAGAAGA
	gTSC1_20A	AGTTGGGGAACCTCTGTCCTA
Exon 19	gTSC1_21S	CAGAATCTTTCTGCAGCATCC
	gTSC1_21A	CAGCACCAAAAACATGAACCT
Exon 20	gTSC1_22S	CCATTATGTCAGGGACTGTGAA
	gTSC1_22A	TAGCTGGACCACGGAGTAGTG
Exons 21 + 22	gTSC1_23S	GCTTGGGGATAGATTTCAAGG
	gTSC1_23A	ACACGGAGTGAGCTGAGTGTT
	gTSC1_24A	TGCAGCTGTCCTCTGAAAGAT
	gTSC1_24S	GTCAAACTCCAGGCAAGGTAA
Exon 23	gTSC1_25S	CATATGGCCACAGGAAGTGTT
	gTSC1_28A	CCGTCCCATTTCCACACATG
	gTSC1_26A	CAGAAAGGCTACTGGTCATGC
	gTSC1_26S	GGGAGACGACTATGGGAGAAG

**Table A2:** PCR and Sanger sequencing primers for the *TSC2* gene.

Targeted Segment	Name	Oligonucleotide Sequence (5′–3′)
Promoter + exon 01	gTSC2_1S	CGAGGACAGCAAGTTCACTG
	gTSC2_1A	GTTTGCCGTCTCTCCTCTACC
	gTSC2_2A	GAGCTTGCTGGGAGTTGTAGTT
	gTSC2_2S	CTACCTGCTGCAGCCTCTCT
Exon 02	gTSC2_3S	GGTAGAGGAGAGACGGCAAAC
	gTSC2_3A	AAGTGTGCCTGAACCAGGTC
Exon 03	gTSC2_4S	CGGCTCGTCAAGTGAATCTT
	gTSC2_4A	GTCAGCTGTCAACCATGTTCC
Exon 04	gTSC2_5S	TGAGACTGTCCCATGACTTCC
	gTSC2_5A	AGGGCAAAACAACACCGTAG
Exon 05	gTSC2_43S	CCTGCCCTGTACAATGCTGA
	gTSC2_43A	CAAGCCCCAGAGACTCACAG
Exon 06	gTSC2_44S	GATCCTAGTGTCCGTGCGTAG
	gTSC2_44A	CGGAGCTGAACTTAGGACCAT
Exon 07	gTSC2_8S	GCTCTCATCTGATGTCTTGGTT
	gTSC2_8A	GTCATTGATGCTGTCATCCAC
Exon 08	gTSC2_9S	GTCCCCCATGTAAGTCAGGAT
	gTSC2_9A	ATCTCCTCCCAAAGACAGAGG
Exon 09	gTSC2_10S	CTGTCTCCCATGAATGGTTGT
	gTSC2_10A	GGCTAAGTAGTTGGGGAGCAC
Exon 10	gTSC2_45S	GTGTTACTGCTGGCCTCTGT
	gTSC2_11A	CAGCTCACTGCACACAGAAAC
Exon 11	gTSC2_12S	GGATTCAGTTGCTGGTCTGTC
	gTSC2_12A	ACTAATGCGGTCCTCCAAAGT
Exon 12	gTSC2_46S	CCTCTGGTGCCAAGTCCATG
	gTSC2_45A	CCCTAAGCTGAGTGTTCCTGG
Exon 13	gTSC2_14S	CAGTTTCCTCCCACCTGTGT
	gTSC2_14A	GGAGCATCTCTCCAGACGAC
Exon 14	gTSC2_15S	GTGCTAGCTTGCTTTCCAGTC
	gTSC2_15A	AGACTGGCTGAAACGAACTCA
Exon 15	gTSC2_16S	GCTGCTCCTTGTGAGTTGTG
	gTSC2_16A	ACTGTGCAGAAACCAAAATGC
Exon 16	gTSC2_17S	CTCAGAACCATGAGCCTGTGT
	gTSC2_17A	AGCGTGTGCTACTGGTATGCT
Exon 17	gTSC2_18S	GTTGATGACTGCCCTGATGAT
	gTSC2_18A	TTAGAGCGACAAGCCACAGAT
Exon 18	gTSC2_19S	CAGAGTCCTGTTCAGCCTGTC
	gTSC2_19A	GAAGCAAGAGAAGCAGCTGAG
Exon 19	gTSC2_20S	CTACATGTACGCGGGACCTC
	gTSC2_20A	GCCTTCTGGACCCTAGAGACA
Exon 20	gTSC2_21S	GTGCCCTACTCCCTGCTCTT
	gTSC2_46A	GCTCGCAGTCTTTTGGGGAA
Exon 21	gTSC2_47S	GTGTGTTACTTGGCAGGCAC
	gTSC2_22A	GTGGACAGGGAACACTGGAT
Exon 22	gTSC2_23S	GAGTCTGCTCGGGTAGCTCA
	gTSC2_23A	ACCTGAGCTCCTGAAGTCACA
Exon 23	gTSC2_24S	TCACGGATCACACAAATGGTA
	gTSC2_24A	GAGCCCACCTTAGTGATGAAA
Exon 24	gTSC2_25S	CGCACCTCTACAGGAACTTTG
	gTSC2_25A	GAGTGAGCACACCCAGACAGT
Exon 25	gTSC2_26S	TCATCACTAAGGTGGGCTCAG
	gTSC2_26A	AACCCCCAATTCCACAAGTAG
Exon 26	gTSC2_27S	ACCCACACACGTTTAATTTGC
	gTSC2_27A	GAATACGAAAAGGCCAAAACC
Exons 27 + 28	gTSC2_28S	AATGTGGTCCACGTGATTCTC
	gTSC2_28A	GACTTAGTCCCCAGGCTGGTA
Exon 29	gTSC2_29S	CGCTCCCTGTCTTCTAGGTCT
	gTSC2_29A	CAGAGAAGGGCTCCAGGACT
Exon 30	gTSC2_48S	CTTGAGGCTGGTGGTTTTGC
	gTSC2_47A	AGAGGGCCAAGTCTGCAATC
Exon 31	gTSC2_31S	TGAGGGGTGCAAAGAGTAGG
	gTSC2_31A	GGAGAACAATGGTGCTGAGG
Exon 32	gTSC2_32S	GACGTCTATTCACGGGAGGA
	gTSC2_32A	CTAAACAGCTGCCACCCATC
Exon 33	gTSC2_33S	GTTACGAGGGCTGGTTTCAG
	gTSC2_33A	ACACTGCGTGAGCAGAGGTAT
Exon 34	gTSC2_34S	ATACCTCTGCTCACGCAGTGT
	gTSC2_34A	AGCTGCAGGAACACGAAACT
	gTSC2_35A	CTCTTTAAGGCGTCCCTCTCT
	gTSC2_35S	CTGTGGACCTCTCCTTCCAG
Exon 35	gTSC2_36S	AGCCTCCAATGCAGAGAAAGT
	gTSC2_36A	GTGTCGTATGATGGGATCTGG
Exon 36	gTSC2_37S	TGTTCCTGCAGCTCTACCATT
	gTSC2_37A	TGTCAGCTCACTGACCAACAG
Exon 37	gTSC2_38S	GAGGGAAGAGAGGGAGTCAAG
	gTSC2_38A	GGCACCTCCTGATTACTCCA
Exon 38	gTSC2_39S	CTCCCATCCAGTCCTGCTAC
	gTSC2_39A	TCTGCACTTGCCAGTTACTCC
Exons 39 + 40	gTSC2_49S	CAGAGGGGAAAGTTCAGGGG
	gTSC2_40A	GTAGATATCGGTGGGGTTGGA
Exon 41	gTSC2_41S	AAGTCTCCCCAGACATGGAG
	gTSC2_41A	CACAAACTCGGTGAAGTCCTC
Exon 42	gTSC2_42S	CCGATATCTACCCCTCCAAGT
	gTSC2_48A	CTTCTAGAGCCTCGACACCC

**Table A3:** Nextera Flex capture coverage per individual. Total reads, mean reads per nucleotide, granular, and target region covered are shown.

ID	Coverage.		Granular			Target Region Covered (%)	
	Total	Mean	Third_Quartile	Median	First_Quartile	>10 Reads	>20 Reads
14	193108927	164.54	215	160	110	98.40%	97.40%
34	206241477	175.73	230	168	114	98.50%	97.60%
46	51912227	205.33	252	198	151	99.50%	99.10%
50	34975423	138.34	173	122	84	99.50%	98.90%
53	60450935	239.1	294	226	170	99.90%	99.40%
56	47703937	188.68	235	169	119	99.70%	99.30%
60	35594649	140.79	175	126	89	99.60%	99.20%
63	43388916	171.61	215	151	106	99.70%	99.30%
64	56625909	223.97	278	218	165	99.40%	99.00%
65	64631748	255.64	314	249	190	99.40%	99.10%
66	45574283	180.26	225	178	134	99.20%	98.70%
82	41660923	164.78	205	162	122	99.20%	98.70%
87	56281945	222.61	274	219	167	99.40%	99.00%
100	52273457	206.76	255	203	154	99.40%	99.00%
104	48197580	190.63	235	180	136	99.60%	99.20%
113	57003770	225.46	277	215	163	99.70%	99.30%
116	41915283	165.79	207	157	117	99.60%	99.10%
117	40770943	161.26	200	159	120	99.10%	98.50%
118	46434270	183.66	226	179	136	99.40%	99.00%
119	48891382	193.38	241	192	144	99.20%	98.80%
123	65664401	259.72	335	246	170	99.50%	99.20%
124	60099947	237.71	307	221	152	99.50%	99.10%

**Table A4:** Quantitative PCR primers for *TSC1*, *TSC2*, *GAPDH*, and *SPACA9* genes.

Segment	Name	Oligonucleotide Sequence (5′–3′)
*TSC1*		
Exon 01	qPCRgTSC1_1S	AGGGACTGTGAGGTAAACAGC
	qPCRgTSC1_1A	AGGAAGCCCCCATAAAAAGGAG
Exon 17	qPCRgTSC1_4S	CAGATGAGATCCGCACCCTC
	qPCRgTSC1_4A	AGCTGCTGCTTTGATCACCT
*TSC2*		
Exon 12	qPCRgTSC2_2S	TCCATGACCTGTTGACCACG
	qPCRgTSC2_2A	CGCACATCTCTCCACCAGTT
*PKD1*		
Exon 45	qPCRgPKD1_1S	GGCTCTCTACCCTGTGTCCT
	qPCRgPKD1_1A	CGGAGAATAACAGCCCCCAG
Exon 46	qPCRgPKD1_2S	TAGGTGTGGTGGCGTTATGG
	qPCRgPKD1_2A	CTCTGGGGTGATGAGAGTGC
*GADPH*		
Intron 01	gGAPDH_1S	GCTCCCACCTTTCTCATCC
	gGAPDH_1A	CTGCAGCGTACTCCCCAC
*SPACA9*		
Exon 04	qPCRgSPACA9_1S	GAGGCCCGGAACCACTAC
	qPCRgSPACA9_1A	GAGTGGGCGATCCATTCTTTC
	qPCRgSPACA9_2S	GAGGCCCGGAACCACTAC
	qPCRgSPACA9_2A	GAGTGGGCGATCCATTCTTTC

**Table A5:** Novel non-pathogenic exonic variants identified in *TSC1* and *TSC2* (LovD and dbSNP databases were accessed in June/2024).

Segment	Variant	Variant Type	Highest Reported Frequency/Database
*TSC1*			
3′UTR	c.*1G>A	3′ UTR	NR
3′UTR	c.*191dup ^a^	3′ UTR	NR
3′UTR	c.*1362C>T	3′ UTR	NR
*TSC2*			
Exon 30	c.3431T>A (p.V1144E)	Missense	NR

NR: not reported. ^a^ Identified in two patients.

**Table A6:** Novel non-pathogenic upstream and intronic variants identified in *TSC1* and *TSC2* (LovD and dbSNP databases were accessed in June/2024).

Segment	Variant	Variant Type	Highest Reported Frequency/Database
*TSC1*			
5′ UTR	c.-606_-605delGCinsTG	5′ UTR	NR
5′ UTR	c.-568C>T	5′ UTR	NR
5′ UTR	c.-282_-281insC	5′ UTR	NR
Intron 11	c.1142-26_1142-25del	Intron variant	NR
*TSC2*			
Intron 07	c.648+12C>A	Intron variant	NR
Intron 12	c.1257+109dup	Intron variant	NR
Intron 15	c.1599+85_1599+87del	Intron variant	NR
Intron 15	c.1599+282_1599+283inCTGGGG ^b^	Intron variant	NR
Intron 21	c.2356-63A>G	Intron variant	NR
Intron 25	c.2837+93G>A	Intron variant	0.000008
Intron 30	c.3610+46dup ^a^	Intron variant	NR
Intron 34	c.4493+110G>A	Intron variant	NR
Intron 36	c.4663-56C>G	Intron variant	0.000004
Intron 37	c.4850-133C>T ^a^	Intron variant	NR

NR: not reported. ^a^ Identified in two patients. ^b^ Identified in three patients.
